# Ovine epididymitis – a neglected notifiable disease in Germany: a case report

**DOI:** 10.1186/s12917-026-05673-8

**Published:** 2026-06-27

**Authors:** Hanka Brangsch, Martin Peters, Elisabeth M. Liebler-Tenorio, Martina Poppe, Udo Moog, Roland T. Ashford, Falk Melzer

**Affiliations:** 1 Friedrich-Loeffler-Institut, Institut für bakterielle Infektionen und Zoonosen, Jena, Germany; 2 Chemisches- und Veterinäruntersuchungsamt Westfalen (CVUA Westfalen), Arnsberg, Germany; 3 Friedrich-Loeffler-Institut, Institut für molekulare Pathogenese, Jena, Germany; 4 Kreisverwaltung Soest, Veterinärdienst, Soest, Germany; 5 Thüringer Tierseuchenkasse AdöR, Tiergesundheitsdienst, Jena, Germany; 6 Department of Bacteriology, Animal and Plant Health Agency (APHA), Addlestone, UK

**Keywords:** Ovine contagious epididymitis, *Brucella ovis*, Sheep, Whole genome sequencing, SNP typing, Immunohistochemistry, Germany

## Abstract

**Background:**

Ovine contagious epididymitis is an infectious disease caused by *Brucella ovis*. Its occurrence in EU member states must be reported to the relevant authorities. Although it is considered endemic in parts of Europe and other sheep-rearing regions world-wide, the disease is rarely reported. Hence, detailed information about the epidemiological situation and transmission events in Europe remains scarce. Unlike other forms of *Brucella* infection, *B. ovis* is not transmissible to humans, making it primarily an economic and animal health problem. Nevertheless, the same control measures can be applied for outbreaks of ovine epididymitis and livestock brucellosis caused by other *Brucella* species in Germany.

**Case presentation:**

In 2024 *B. ovis* was isolated from two stillborn lambs found dead in Germany. Immunohistochemistry detected *Brucella* antigen in the pulmonary airways of both lambs. Additionally, one lamb exhibited necrotizing hepatitis of the liver. One *B. ovis* isolate was sequenced and genotyped, revealing genomic similarity to other European *B. ovis* strains. Two rams from the same flock tested serologically positive for *B. ovis*, but none of the ewes with a history of abortion did. There was also no increase in the rate of abortions or stillbirths in this flock. Following the slaughter of two of the tested rams in 2025, the testicles and epididymides of both animals were examined and *B. ovis* could be detected by immunohistochemistry and PCR in some of the tissues, although no isolate could be obtained.

**Conclusions:**

Little is known about the prevalence of ovine epididymitis in Germany. The case presented here suggests that the disease is likely underdiagnosed and therefore underreported. Although the pathogen is not zoonotic, awareness of *B. ovis* infections should nevertheless be raised to improve animal welfare.

**Supplementary Information:**

The online version contains supplementary material available at 10.1186/s12917-026-05673-8.

## Background

*Brucella* infections in small ruminants may be caused by different *Brucella* species, namely *B. melitensis*, *B. ovis* and rarely *B. abortus* [1]. The main causative agent worldwide is *B. melitensis*, whereas the epidemiological situation regarding *B. ovis* is less well-understood. *B. ovis* is a Gram-negative bacterium that occurs naturally in rough form and has a narrower host range than other classical brucellae. Its main reservoir is sheep. The bacterium causes a disease referred to as ovine contagious epididymitis. In contrast to *B. melitensis* and *B. abortus*, zoonotic *B. ovis* infections are not reported. It can be assumed that *B. ovis* is present in almost all sheep-raising regions in the world. First reported in Australia and the USA in the 1950s [2, 3], *B. ovis* has been found in various countries on all inhabited continents, e.g. New Zealand [4], Australia [5], Saudi Arabia [6], Argentina [7], Brazil [8, 9], Canada [10], USA [11], and South Africa [12]. In Europe, *B. ovis* has been reported from many countries across the continent, including in France [13], Spain [14], Austria [15], Switzerland [16], Italy [17, 18], Croatia [19], Serbia [20] and Bulgaria [21]. According to Commission Implementing Regulation (EU) 2018/1882, the European Commission lists ovine contagious epididymitis among the diseases of priority [22]. As a result, European Union (EU) member states must establish a surveillance system and report every occurrence of the pathogen. Furthermore, animals must be tested before export within the EU to prevent spread of the disease (EU Animal Health Law).

While most reports are from sheep, *B. ovis* can also infect other ruminants, notably goats and deer [9, 21, 23]. Transmission usually occurs via the venereal route, with the presence of the pathogen demonstrated in genital secretions, semen and urine [10, 17, 24]. In rams, the infection can manifest as scrotal asymmetry, unilateral swelling of the epididymal tail, testicular atrophy, diffuse fibrosis, tunica vaginalis thickening, and epididymal abscesses [17, 20]. Some of these symptoms may be detectable by scrotal palpation one to two months after infection, whereas shedding of the bacteria may begin earlier [4]. Epididymitis in rams can be detected during a breeding soundness examination, i.e. a physical examination and semen evaluation by microscopy prior to mating. However, the infection often progresses subclinically, meaning that infected animals do not show any, or only poorly pronounced, symptoms [10, 20]. This complicates the timely detection and eradication of ovine contagious epididymitis. Symptoms in ewes include placentitis, stillbirth and an increased abortion rate, among others. Although *B. ovis* can be shed in the milk, the infection is not typically transmitted to lambs [24]. Ewes can clear the infection during subsequent pregnancies and do not necessarily pass it on to rams [24].

The primary method for detecting infection with *B. ovis* is serological testing, which can yield positive results as early as 36 days post infection [4]. For this purpose, the complement fixation test (CFT) is the only internationally recognized standardized test [25], although the indirect enzyme-Iinked immunosorbent assay (I-ELISA) is more sensitive and can therefore also detect early-stage infections [4, 9, 13, 20].

Although several publications report the isolation of *B. ovis* from sample material [13, 20], few studies have used whole genome sequencing (WGS) for epidemiological investigation [18, 26]. Particularly in Europe, there is a lack of comprehensive studies on the epidemiology of *B. ovis*, despite the above-mentioned reports concerning its widespread occurrence.

In Germany, ovine contagious epididymitis is a notifiable disease that must be investigated by the state veterinary investigation offices. In the event of a suspected *B. ovis* outbreak in sheep, the competent authority may order the measures prescribed in the German Brucellosis Ordinance for sheep, to the extent necessary to control the disease (BrucelloseV). This includes serological testing of all animals over six months old in the affected flock and culling of seropositive animals, as well as disinfecting potentially contaminated paddocks and pastures. Animals from these flocks must not be moved to other flocks, shorn or mated. However, it is at the discretion of the responsible office whether these measures are demanded. Artificial insemination currently plays a relatively minor role in the German sheep breeding industry and animals must be tested seronegative for *B. ovis* beforehand (EU 2020/686).

In Germany, there are about 19,870 sheep farms [27, 28] and almost half of them keep less than 20 sheep. In 2024, there were 9,670 farms with flocks of at least 20 animals (https://www-genesis.destatis.de/datenbank/online/statistic/41314/table/41314-0001; accessed: 2025/10/28). Only around 30% of sheep farms operate as full-time enterprises, while a further 40% undertake additional agricultural activities and the remaining farms are small-scale or hobby farms. The main purpose of sheep farming is landscape management and meat production, whereas milk and wool production are negligible [27].

Despite reports of *B. ovis* occurring in neighbouring countries, including France, Switzerland and Austria, there are no reports on the prevalence of ovine contagious epididymitis in Germany. In the German Animal Disease Reporting System, which is the official platform for reporting outbreaks of notifiable animal diseases, only a single outbreak of *B. ovis* has been reported in the last 15 years. This outbreak will be the subject of the current study, in which we present a case of ovine contagious epididymitis in stillborn lambs and two rams held in North Rhine-Westphalia, Germany. The lambs showed histological lesions and *B. ovis* was isolated from various samples, including liver and lung tissue. One of the isolates was sequenced and compared to other available *B. ovis* strains. One of two seropositive rams exhibited macroscopic lesions in the epididymis, but *B. ovis* could not be isolated.

## Case presentation

In January 2024, two stillborn lambs were found by passersby in North Rhine-Westphalia, Germany, on a pasture where a flock of sheep had previously been grazing. The responsible veterinary office was immediately notified. Due to the unclear cause of death and possible violation of the carcass disposal law, the lambs were sent for postmortem examination. At the time of the finding, the farm in question had approximately 2,300 ewes and rams as well as around 700 lambs. Flocks were frequently reorganized. Ewes were brought into the pen to lamb and as soon as the ewes and lambs were settled, they were returned to the extensive pasture.

The stillborn lambs underwent a complete postmortem and microbiological examination to determine potential infectious causes of abortion. One lamb showed a mild acute necrotizing hepatitis. Both lambs had atelectatic lungs. In the pulmonary airways, particularly in the alveoli, aspirated amniotic fluid was present in the form of amorphous material mixed with cellular debris.

Immunohistochemistry was performed on formalin-fixed paraffin-embedded (FFPE) sections of brain, lung, heart, spleen and liver by indirect immunoperoxidase reaction using orb4727 (Biorbyt/Biozol, Eching) as primary antibody and Zytochem Plus HRPPolymer anti-rabbit (Zytomed systems/Biozol, Eching) as secondary antibody, with antigen retrieval by microwave treatment in citric acid buffer (pH 6.0), and 3-amino-9-ethylcarbozole as chromogen, with hemalaun as counter stain. As positive control, sections of muscle spiked with *B. ovis* type strain 63/290 (ATCC 25840) were used. As negative control, sections of the epididymis from a healthy ram were used and for each section from the infected ram, the specific primary antibody was replaced with an antibody against an unrelated antigen (*Brachyspira hyodysenteriae*).

**Fig. 1:**
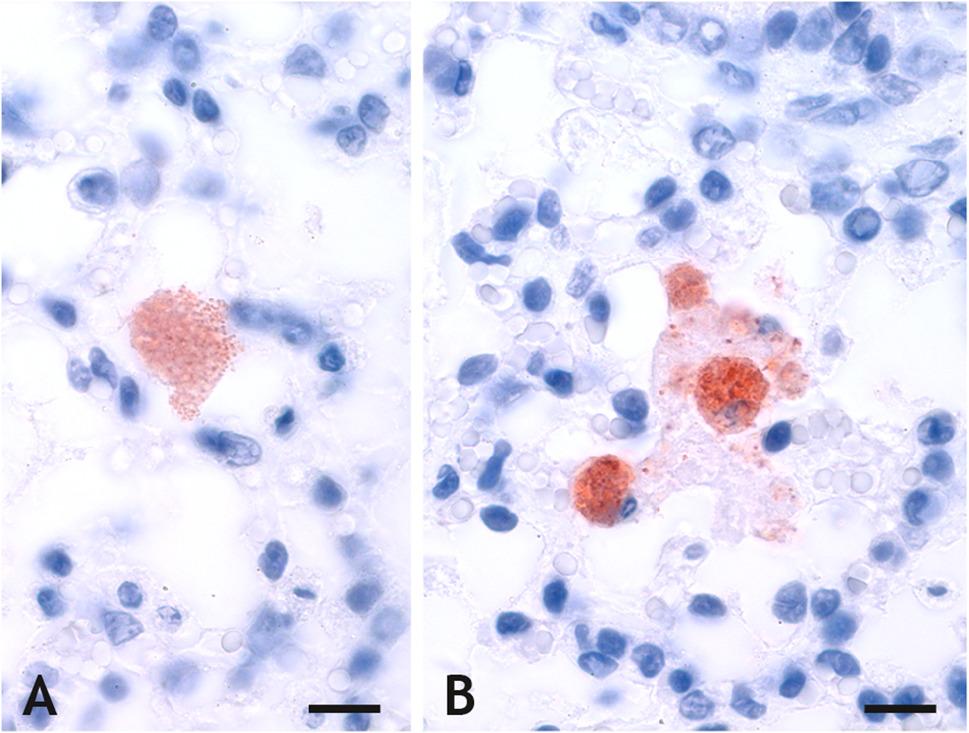
Detection of *Brucella* antigen by immunohistochemistry in the stillborn lambs. **A**, **B ***Brucella* antigen is present in aspirated allantoic fluid in pulmonary airways, extracellular, as clusters (**A**) and intracellularly (**B**). Bars = 10 μm

Using immunohistochemistry, *Brucella* antigen was detected extracellularly as clusters of coccoid to short rod-shaped bacteria and within cells of the aspirated amniotic fluid in the pulmonary airways of both lambs (Fig. 1). In the liver of the lamb with necrotizing hepatitis, faint staining of a few bacteria was observed in the necrotic areas, and in the macrophages within the periportal infiltrates as well as within the sinusoids. *Brucella* antigen was not detected in the hearts or brains of either of the lambs, nor in the liver of the lamb without hepatic lesions. *B. ovis* antigen was detected in all positive control sections. No reaction product was observed in any of the negative control sections.

From both lambs, *Brucella* were isolated from stomach, liver and lung on *Brucella* agar I (BRAG) (Supplementary Table S1) under microaerophilic conditions at 37 °C for 5 days. Colonies were picked and subjected to matrix-assisted laser desorption/ionization time of flight mass spectrometry (MALDI-TOF) (Bruker Daltonics GmbH, Bremen, Germany) yielding a high score for *Brucella melitensis*. For further identification, the isolates were sent to the German National Reference Laboratory for Bovine, Porcine, Ovine and Caprine Brucellosis (Friedrich-Loeffler-Institut).

DNA was isolated using the High Pure PCR Template preparation kit (Roche Molecular Systems, Pleasanton, CA, United States) and the identity of the isolates as *B. ovis* was confirmed by BruceLadder PCR and AMOS PCR as described by Mayer-Scholl et al. [29], Lopez-Goni et al. [30] and Bricker et al. [31], respectively. These conventional PCRs enable differentiation of *Brucella* species based on the pattern generated by the products in the subsequent agarose gel electrophoresis. The patterns of the investigated strains were identical to that of the *B. ovis* positive control (*B. ovis* strain 63/290). No bands were observed in the negative controls.

The DNA of one isolate (24RB27234) was sequenced on an Illumina Miseq (Illumina Inc., San Diego, CA, United States) using the Illumina DNA Prep kit for library preparation. Sequencing data quality was assessed, the genome assembled and the assembly quality controlled as described elsewhere [32]. Raw reads were deposited with the European Nucleotide Archive under BioProject number PRJEB98849.

**Fig. 2:**
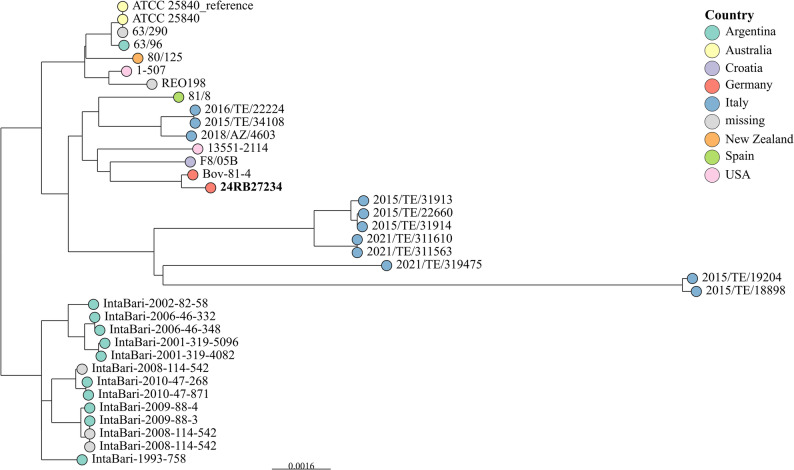
Maximum likelihood tree based on the cgSNP alignment of international *B. ovis* strains (see Supplementary Table S2). The bar indicates base substitutions per site

The genome assembly of 24RB27234 comprised 3,237,750 bp in 55 contigs and showed a GC content of 57.22%. The isolate belonged to multi-locus sequence typing (MLST-9) sequence type 13 (ST13). For comparing 24RB27234 to other field strains, available *B. ovis* short-read sequences were downloaded from NCBI’s Short Read Archive (accessed on 28.01.2026) (Supplementary Table S2) and subjected to core genome single nucleotide polymorphism (cgSNP) analysis using Snippy v4.6.0 (https://github.com/tseemann/snippy) with *B. ovis* ATCC 25,840 (GCF_000016845.1) as reference genome. Additionally, data of a historic German *B. ovis* strain (Bov-81-4 isolated from sheep in 1981) was provided by the Animal and Plant Health Agency (APHA), United Kingdom. The cgSNP alignment was analyzed by maximum likelihood analysis using RAxML v8.2.12 [33] and the phylogenetic tree was visualized by Microreact [34]. Isolate 24RB27234 differed by 142 to 595 cgSNPs from non-German isolates, which originated primarily from Argentina and Italy (Fig. 2). However, a comparatively high level of similarity (32 cgSNPs difference) was found with the historic German isolate Bov-81-4 from 1981. Both German isolates differed in 142 and 128 cgSNPs, respectively, from strain F8/05B, isolated in Croatia in 2005. This analysis of the limited available dataset confirmed the isolate’s identity as *B. ovis* and suggested the existence of an endemic German *B. ovis* lineage, that has persisted for decades.

Following confirmatory tests at the reference laboratory, the outbreak was officially reported in the German Animal Disease Reporting System. Three rams (rams A, B and C) were considered as potential sires of the stillborn lambs, one of which showed clinically enlarged testicles (ram C). Serological testing was performed on these three rams and three ewes, which had recently experienced abortions, using the complement fixation test (CFT) according to the EU Reference Laboratory for Brucellosis protocol (https://sitesv2.anses.fr/sites/default/files/SOP%20Brucellosis%20EU-RL%20B.%20ovis%20CFT_Rev1_vf.pdf; accessed 02.01.2024). Two rams (rams A and C) were positive by CFT for *B. ovis* antibodies, whereas the ewes and ram B were negative (Table 1). No increase in the rate of abortions or stillbirths was observed in either the overall population or the affected flock (approximately 150 ewes and three rams). The rams were capable of mating and had successfully impregnated ewes. The responsible authority therefore made an assessment based on a case-by-case analysis of the individual circumstances. The farmer was advised to promptly cull the serologically positive rams as a preventive measure, or at least to isolate them and prevent them from mating. Consequently, the animals were no longer used for breeding and were kept only in fattening flocks. In accordance with the German Brucellosis Ordinance, no further measures were taken and the outbreak notice was lifted.

**Table 1:** Presentation of ovine contagious epididymitis in three rams from a flock in Germany. For CFT, the UI/ml values of positive samples are given – the threshold for a positive result is ≥ 50 IU/ml

Enlarged testicles	Ram A	Ram B	Ram C
	no	no	yes
Necropsy	without macroscopic and pathohistological lesion	swollen right epididymis, pyogranuloma	not performed
CFT	2024: positive (74 IU/ml) 2025: negative	2024: negative 2025: suspicious (44–53 IU/ml)	2024: positive (125 IU/ml)
ELISA (2025)	positive	positive	not tested
PCR (2025)	positive (right testicle, right epididymis)	positive (both epididymis’, pyogranuloma)	not tested
Immunohistochemistry	No detection of *B. ovis*	*B. ovis* detected in tissue	not performed
Remark	slaughtered, April 2025	slaughtered, April 2025	deceased and disposed of without further investigation

**Fig. 3:**
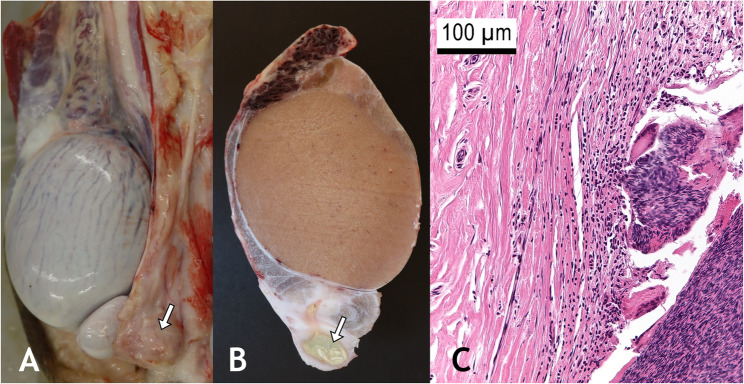
Presentation of ovine infectious epididymitis in ram B. **A**, **B **swollen epididymal tail and pyogranuloma (white arrow) next to the vaginal process. **C **granulomatous inflammation with multinucleated giant cells within the epididymis

In April 2025, 15 months after the stillbirths, the fresh scrotums containing the testicles of two slaughtered rams originally tested in 2024 (ram A and ram B) were submitted for pathomorphological and bacteriological examination (ram C having died and been disposed of without further investigation, without notifying the authorities). Table 1 provides an overview of the results of the tests performed. In both rams, the testes were symmetrical. In ram B, a pyogranuloma was located next to the vaginal process of the right testicle, in intimate contact with the swollen right epididymis (Fig. 3A, B). To sample testicles, epididymides and pyogranuloma independently for bacteriology and PCR, sterile biopsy punches (Ø 8 mm, Kruuse, Langeskov, Denmark) were used on the frozen cut surfaces.

Histological examination revealed a spermatic cyst caused by rupture of the epididymal duct in ram B, with a granulomatous inflammation (Fig. 3C), a fistulous tract, perforation of the vaginal process and a granuloma in the swollen epididymis.

**Fig. 4:**
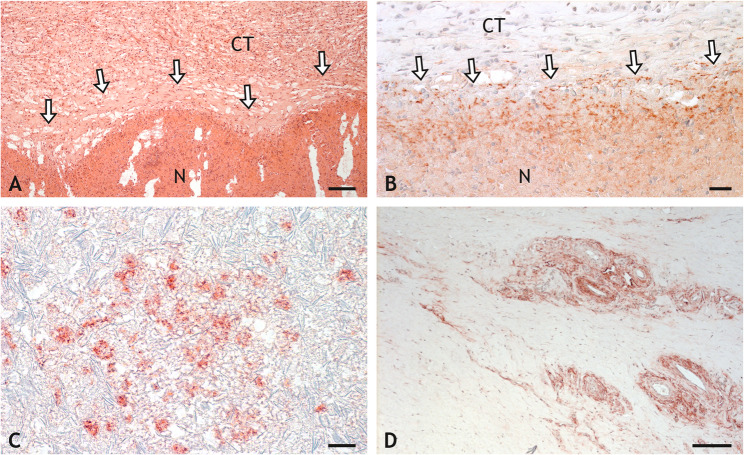
Pyogranuloma of ram B extending into the vaginal process. **A** Necrosis (N) demarcated by a thin layer of inflammatory cell (white arrows) surrounded by connective tissue (CT). **B ***Brucella* antigen in cells of the inflammatory infiltrate (white arrows) demarcating the necrosis (N) from the connective tissue (CT). **C** Clusters of *Brucella* antigen between sperm cells within the necrosis. **D** *Brucella* antigen in cells of the perivascular infiltrate and vascular walls in the surrounding connective tissue. Bar in A, D = 100 μm, in B = 20 μm, in C = 10 μm

*Brucella* antigen was detected in ram B: in the granuloma with central necrosis in the epididymis, as well as in the pyogranuloma extending into the processus vaginalis (Fig. 4). In both locations, *Brucella* antigen was present multifocally in the necrotic debris, between sperm cells and within macrophages in the inflammatory infiltrate surrounding the necrosis. Multinucleated giant cells were rarely positive. *Brucella* antigen was also found in the cytoplasm of macrophages in perivascular infiltrates, as well as in the walls of lymphatic vessels and small blood vessels within the thick, vascularized fibrous connective tissue capsule surrounding both lesions. *Brucella* were not detected in the testicles.

*Brucella* could be detected by conventional PCR targeting *bscp31* [35], a gene that is specific to all *Brucella* species, within the pyogranuloma, the inflamed right epididymis and the left epididymis of ram B, as well as in the right testicle and right epididymis of ram (A) Additionally, the blood plasma and serum of both rams was tested by ELISA (IDEXX *Brucella ovis* Ab test, IDEXX Laboratories, Inc., Maine, USA). All blood samples yielded positive results for *B. ovis* in the ELISA, whereas CFT yielded a negative result for ram A and a suspicious result for ram B. Despite several attempts, no *B. ovis* isolate could be obtained from any of the tested materials. For these isolation attempts, several media (listed in Supplementary Table S1), solid and liquid, at the conditions described above, were used, in accordance with the WOAH Terrestrial Manual. Cultures were incubated up to 16 days, but no growth was observed. This failure of isolation could not have been caused by a problem with the cultivation media, as these were tested beforehand using a control strain (*B. ovis* 63/290 (ATCC 25840)) to ensure their quality and suitability.

## Discussion and conclusions

The impact of *B. ovis* infection in sheep in Germany is currently unknown. Although *B. ovis* infections have been detected in neighboring countries such as Austria and Switzerland [15, 16], to our knowledge, this is the first report of *B. ovis* in a flock of sheep in Germany. Since the disease is primarily an animal health issue and sheep farming constitute a small proportion of the overall farming activities, the economic impact in Germany is likely to be minimal. This could explain why the disease may be underreported not only in Germany, but also in other countries. However, the frequently observed reduction in semen quality [10, 13] could reduce breeding success, potentially resulting in losses for individual owners. In the reported case, no defect in mating success was observed, despite *Brucella* being detected between the sperm in one ram. Nevertheless, the owner was reluctant to kill the seropositive animals and he could not be legally compelled to do so. In countries with higher prevalence and greater economic impact, for example Serbia, sheep owners are more aware of *B. ovis* infections and remove potentially infected animals [20]. According to the EU Animal Health Law, there is no legal basis for the authorities to intervene unless animals are being moved within or exported from the European Union. As there were to be no movement or export of sheep from the flock in question, the authorities had no legal basis under applicable EU law to take action. The German Brucellosis Ordinance states that no measures need to be taken in the event of a *B. ovis* infection, except to the extent necessary to combat animal diseases. Due to the multiple mixing of flocks on the affected farm before the outbreak was detected, and because the rams had also been transferred to another flock (fattening ram lambs) in the meantime, further sampling of animals was not carried out. Only the slaughter or isolation of the rams was recommended.

In recent decades, various countries (e.g. Australia, Austria, Croatia) have introduced eradication programs to combat ovine contagious epididymitis [5, 15, 19, 36]. In the 1980s and 1990s, Australia and Austria, respectively, implemented testing and culling strategies for rams based on serological tests and examinations for clinical symptoms [5, 15]. If a positive animal was found, all rams of the flock had to undergo CFT or ELISA testing at two consecutive time points at least four weeks apart. Furthermore, rams intended for breeding or auctions must be serologically certified as negative [15]. In Tyrol, Austria, sheep owners received financial reimbursement for culled animals and the state supported the costs of serological examinations. Following these approaches reduced the seroprevalence of *B. ovis* infection, but did not eradicate the disease. The same was observed in Croatia, where the disease persisted despite the implementation of an eradication program that comprised culling or castration of infected rams [19]. The only successful eradication program was reported from the Falkland Islands, where a combination of palpation of the external genitalia, serological tests and culling of positive animals was applied. This was done in combination with a voluntary accreditation campaign for *B. ovis*-free flocks [36].

Perhaps the biggest obstacle in eradication campaigns is the inconsistency in disease manifestation. The symptoms observed in the animals investigated in the current study, e.g. enlarged testicles and a testicle abscess, were consistent with other reports. An increase in abortions or stillbirths could be expected when the disease is introduced to a naïve flock or to primiparous females, since ewes can clear the infection during subsequent pregnancies [24]. Based on the material found in the lungs of the lambs in the presented case, it can be assumed that the ewes had a bacterial and/or *Brucella*-induced placentitis. As *Brucella* cells were detected in the semen of the rams, transmission via the venereal route is the most likely explanation. The presence of hepatitis in the lambs is an indication of a systemic spread: from the inhaled material in the lung to the liver.

However, a *B. ovis* infection does not necessarily coincide with an increased abortion rate or decreased fertility [17], in accordance with the findings of the reported case, in which no increase in abortions was observed in the flock. Furthermore, there is a lack of reliable clinical markers, as the animals often do not exhibit pathognomonic clinical symptoms [10] and semen quality may also be normal [37]. Additionally, other bacteria can also cause epididymitis, such as *Actinobacillus seminis* and *Histophilus somni* [38, 39]. Therefore, the infection may not be detected in routine animal examinations, which prevents timely discovery and thus control and eradication. In the reported case, only one of the serologically positive rams exhibited clinical signs, i.e. enlarged testicles, whereas the other can be considered an asymptomatic carrier. Eradication is made more difficult by the fact that these asymptomatic and even seronegative animals can be active shedders of *B. ovis* [10, 13, 37]. This was seen in ram B, which was seronegative but had clinical signs and lesions. Shedding of bacteria is also often intermittent, i.e. not all seropositive animals are culture-positive for *B. ovis* [14]. This is consistent with the unsuccessful attempts to cultivate *B. ovis* from the testicular and epididymal tissue of rams A and B. It has to be noted that *B. ovis* isolation also requires appropriate cultivation media. Various media had been tested in this study: general-purpose nutrient agar supplemented with blood and selective media for *Brucella* spp. Despite prolonged incubation, no brucellae could be isolated from these two rams. Although immunohistology revealed a clear association between lesions and the presence of *Brucella* antigen, the bacteria may only colonize a limited area of tissue. The affected vessels were only positive in the vicinity of the lesions. These could be the starting point for the further spread of the pathogen, as the detection of *Brucella* antigen in the vessels around the lesions in the epididymis indicate a dissemination of the bacteria. Of course, staining does not indicate whether the bacteria are intact and alive. While the molecular methods used here support the presence of the bacterium in the animal, no viable *B. ovis* cells could be detected in the isolation attempts. Therefore, failure to isolate *B. ovis* may indicate the absence of living bacteria, whereas the antigen, i.e. bacterial cell debris, persisted in the tissue.

A coordinated study on the prevalence of ovine contagious epididymitis is required in order to assess the situation and the necessary measures in Germany. Previous studies have shown that, even when *B. ovis* is detected in a flock, only a few animals may be infected, whereas the overall number of affected flocks can indicate a higher prevalence [11].

There is an additional, Germany-specific regulatory obstacle that may impede the investigation of *B. ovis* infections. Although *B. ovis* is non-pathogenic to humans and can therefore be handled in a laboratory of biological safety level 2, a technical regulation (TRBA 466) considers, for historical reasons, all brucellae as zoonotic. Consequently, the majority, including *B. ovis*, must be handled under biological safety level 3 (BSL3) conditions, which require a higher level of containment. Abortion diagnostics performed in the autopsy room, as a non-targeted activity classified as biosafety level 2 (BSL2), are not affected by this, and the primary cultivation of bacteria therefore poses no problem. However, the situation becomes critical if MALDI-TOF analysis indicates the presence of *Brucella*. Thus, in the authors’ opinion, a new definition and official classification of *B. ovis* as a BSL2 agent is desirable in German regulations, as this would facilitate the detection of the pathogen. Furthermore, diagnostic testing would benefit from improving the reliability of MALDI-TOF in differentiating *B. ovis* from zoonotic brucellae.

Genomic investigations of *B. ovis* isolates could help to understand the distribution of the agent. However, due to the limited number of genome sequences currently available, it is difficult to draw conclusions about the genomic diversity and spread of *B. ovis* lineages. It can be expected that certain genotypes are established in restricted geographic regions, as international trade is probably an insignificant factor in the transmission of the disease, since the regulations of the World Organization for Animal Health (WOAH) stipulate testing before the export of sheep.

The present case indicates that infections with *B. ovis* remain underdiagnosed. Farmers may be unaware of the disease and since the financial pressure is not high, a reduction in reproduction rate may not have a significant economic impact. In Germany, revenues from sheep farming largely come from government subsidies, which also serve as compensation for landscape and levee maintenance (personal communication). Thus, a lower reproductive rate has a less significant impact. In this case, it was the persistence of the local authorities that ensured the matter was pursued. Raising awareness of this infection will help to elucidate transmission pathways and prevalence of the pathogen. This could be achieved by implementing a surveillance program for *B. ovis*, not only in Germany, but across Europe.

## Supplementary information

Supplementary Material 1.

Supplementary Material 2.

## Data Availability

The sequencing data generated in the current study is available in the European Nucleotide Archive under project number PRJEB98849.

## Abbreviations

BSL: Biological safety level
CFT: Complement fixation test
ELISA: Enzyme-linked immunosorbent assay
cgSNP: Core genome single nucleotide polymorphism
MALDI: TOF-matrix-assisted laser desorption/ionization time of flight mass spectrometry
PCR: Polymerase chain reaction
WOAH: Word Organization for Animal Health
