# Supplementary material for: Ovine epididymitis – a neglected notifiable disease in Germany: a case report
Source: BMC Vet Res. 2026 Jun 27;22:380. doi: 10.1186/s12917-026-05673-8 (PMC13322093; doi:10.1186/s12917-026-05673-8)
Supplement: Supplementary file 2 — Supplementary Material 2. [file 12917_2026_5673_MOESM2_ESM.docx]

| **Name** | **Composition** |
| --- | --- |
| *Brucella* agar I (BRAG) | 45 g/l Brucella Medium Base (Oxoid, Wesel, Germany), 5% inactivated horse serum, 0.5% defibrinated sheep blood and 2 flasks/l Brucella Selective Supplement (Oxoid, Wesel, Germany) |
| Nutrient agar with calve blood (NGBK) | 35 g/l Nutrient agar (Merck, Darmstadt, Germany), 7.5% defibrinated calve blood |
| *Brucella* agar II (BRUC) | 43 g/l Difco Brucella Agar (BD Diagnostics, Franklin Lakes, USA) |
| *Brucella* selective agar (BRUSEL) | 43 g/l Difco Brucella Agar (BD Diagnostics, Franklin Lakes, USA), 2 flasks/l Brucella Selective Supplement (Oxoid, Wesel, Germany) |
| *Brucella* serum agar (BRUCPF) | 43 g/l Difco Brucella Agar (BD Diagnostics, Franklin Lakes, USA), 5% inactivated horse serum |
| *Brucella* bouillon (BRUCB) | 28 g/l Brucella Broth (BD Diagnostics, Franklin Lakes, USA) |
| *Brucella* selective bouillon (SELBR) | 28 g/l Brucella Broth (BD Diagnostics, Franklin Lakes, USA), 2 flasks/l Brucella Selective Supplement (Oxoid, Wesel, Germany) |
